# Evolutionary Origin of Prolonged Delayed Fertilization in the Fagaceae

**DOI:** 10.1002/ece3.73662

**Published:** 2026-05-13

**Authors:** Takenori Shagawa, Chihiro Myotoishi, Tetsukazu Yahara, Ryosuke Imai, Min Deng, Akiko Satake

**Affiliations:** ^1^ Graduate School of Systems Life Sciences Kyushu University Fukuoka Japan; ^2^ Kyushu Open University Fukuoka Japan; ^3^ The Kyushu University Museum Kyushu University Fukuoka Japan; ^4^ Department of Forest Molecular Genetics and Biotechnology Forestry and Forest Products Research Institute, Forest Research and Management Organization Ibaraki Japan; ^5^ School of Ecology and Environmental Sciences Yunnan University Kunming Yunnan China; ^6^ Department of Biology, Faculty of Science Kyushu University Fukuoka Japan

**Keywords:** ancestral state reconstruction, Bayesian inference, fruiting, leafing, pollination

## Abstract

The temporal organization of flowering, fertilization, and fruiting is a fundamental axis of life‐history evolution in angiosperms. While most species complete fruit development within a single growing season (“1‐year fruiting”), many Fagaceae species delay fertilization and fruit maturation until the year following flowering (“2‐year fruiting”). Despite its ecological prevalence, the evolutionary origins of this strategy and its coordination with other functional traits remain poorly understood. In this study, we investigated the evolutionary origins of the 2‐year fruiting strategy by reconstructing ancestral states on a phylogeny comprising 88 species that represent all eight genera of Fagaceae. Given the observed association of 2‐year fruiting with animal‐pollinated genera and the potential for leaf habit to constrain reproductive timing, both pollination mode and leaf habit may influence the evolution of 2‐year fruiting. To test these possibilities, we further employed phylogenetic comparative analyses to test whether the fruiting trait is evolutionarily constrained by pollination mode (animal versus wind‐pollinated) or leaf habit (evergreen versus deciduous). Our results support a single origin of 2‐year fruiting in the common ancestor of the major clade excluding *Fagus* and *Trigonobalanus*, followed by multiple independent reversions to 1‐year fruiting in lineages including *Castanea*, *Quercus*, and *Castanopsis*. Ancestral‐state reconstructions of pollination mode and leaf habit strongly support entomophily and an evergreen leaf habit as ancestral traits of Quercoideae, predating the emergence of the 2‐year fruiting strategy. Although entomophily and evergreen habit were ancestral in Quercoideae, our correlation analyses indicate that transitions in fruiting strategy did not depend on changes in these traits. These results provide macroevolutionary evidence that extreme extensions of the interval between pollination and fertilization can be evolutionarily stable yet rarely re‐evolve once lost over long evolutionary timescales.

## Graphical abstract

Two‐year fruiting, in which fertilization and fruit maturation are delayed until the year after flowering, occurs in Fagaceae. Ancestral‐state reconstruction across a molecular phylogeny of 88 species representing all genera indicates a single origin of this strategy, followed by multiple reversions to 1‐year fruiting. No evolutionary association was detected with pollination mode or leaf habit, implying long‐term stability of 2‐year fruiting but limited re‐evolvability once lost.

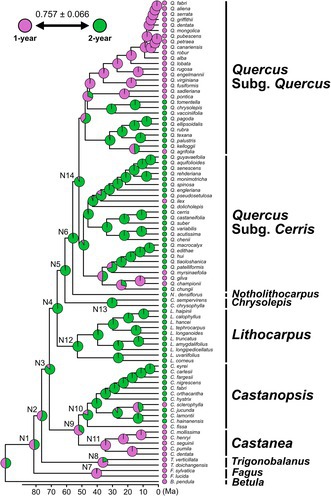

## 1 Introduction

The temporal organization of key reproductive events—flowering, fertilization, and fruit maturation—plays a fundamental role in shaping organismal fitness and underlies phenological and life‐history diversification in angiosperms (Satake et al. 2022; Schwartz 2003). Most angiosperms complete the transition from pollination to fertilization within 24–48 h (Williams 2008; Williams and Reese 2019). However, a subset of taxa has evolved reproductive strategies in which fertilization is substantially delayed, ranging from several days to more than a year after pollination (Sogo and Tobe 2006; Williams 2008; Williams and Reese 2019). These strategies, collectively referred to as delayed fertilization (Sogo and Tobe 2006; Williams 2008; Williams and Reese 2019), represent a striking deviation from the canonical angiosperm reproductive phenology and provide a unique opportunity to explore the adaptive significance of extended reproductive cycles in plants (Deng et al. 2022; Satake and Kelly 2021; Satake et al. 2023; Schoonderwoerd and Friedman 2016; Shagawa et al. 2025; Yao et al. 2023).

**FIGURE 1:**
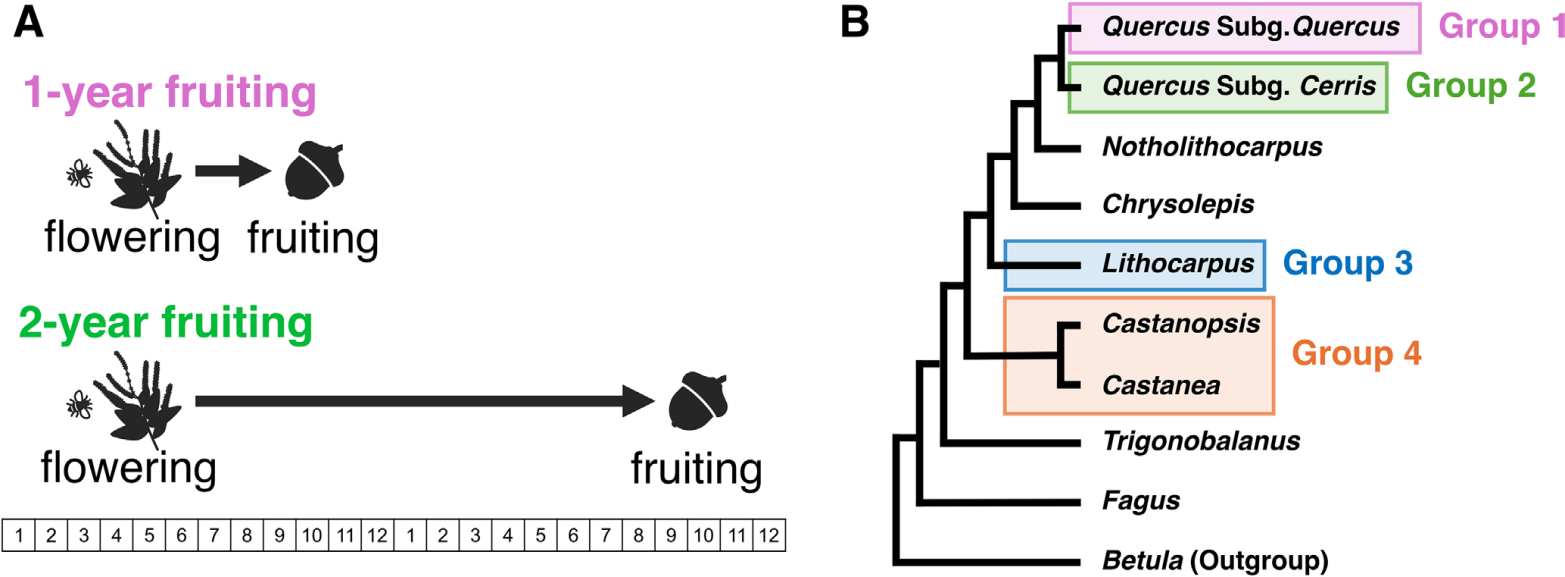
Fruiting strategies in Fagaceae and ancestral state reconstruction of fruiting traits. (A) 1‐ and 2‐year fruiting strategies observed in Fagaceae. In the 1‐year fruiting species, flowering and fruit maturation are completed within a single year, whereas in the 2‐year fruiting species, fruit maturation occurs in the year following flowering. (B) Phylogenetic relationships among Fagaceae genera and the grouping used for ancestral state reconstruction of the fruiting traits. Four groups were defined for the analysis: Group 1 (*Quercus* Subg. *Quercus*), Group 2 (*Quercus* Subg. *Cerris*), Group 3 (*Lithocarpus*), and Group 4 (*Castanopsis* and *Castanea*). The model assumes different transition rates among these groups. Ancestral state reconstruction was performed for the 52 alternative model settings shown in Figure A2, and the optimal model was selected based on the marginal likelihood values calculated for each model.

The Fagaceae, including oaks and beeches, present a particularly striking example of such deviation. In many Fagaceae species, fertilization is delayed until the year following anthesis, resulting in a “2‐year fruiting” strategy in which fertilization and subsequent fruit maturation occur in the year following flowering. In contrast, under the “1‐year fruiting” strategy, the entire reproductive sequence—from anthesis through fertilization to fruit maturation—occurs within a single growing season (Figure 1; Benson 1894; Deng et al. 2022; Sogo and Tobe 2006). Across the family, 1‐ and 2‐year fruiting strategies coexist, with their prevalence differing markedly among genera: all *Fagus* species show 1‐year fruiting, whereas the majority of *Castanopsis* and *Lithocarpus* species exhibit 2‐year fruiting, and *Quercus* shows an intermediate pattern (Araye et al. 2023; Satake and Kelly 2021). Although the 2‐year fruiting with prolonged delayed fertilization has been recognized for more than a century, its evolutionary origins, the frequency and direction of transitions between 1‐ and 2‐year fruiting (Satake and Kelly 2021; Satake et al. 2023; Shagawa et al. 2025).

In relation to pollination mode, 2‐year fruiting tends to be dominant in genera of animal‐pollinated species (*Castanopsis* and *Lithocarpus*), whereas in the wind‐pollinated genus (*Quercus*), 1‐ and 2‐year fruiting are more evenly mixed (Satake and Kelly 2021). Furthermore, in the wind‐pollinated genus *Fagus*, all species exhibit 1‐year fruiting (Satake and Kelly 2021). This pattern suggests that pollination mode may influence the evolution of 2‐year fruiting.

Leaf habit may provide a second, independent axis of constraint. Evergreen species, capable of year‐round photosynthesis, may sustain the prolonged development and overwinter maintenance of ovules or young fruits (Givnish 2002). In contrast, deciduous species—with shorter periods of carbon assimilation and greater risk of carrying immature fruits through winter—may be constrained to complete reproduction within a single growing season (Givnish 2002).

To investigate the evolutionary origin of 2‐year fruiting and test for correlated evolution among fruiting strategy, pollination mode, and leaf habit across the Fagaceae, we combined phylogenetic comparative analyses with a comprehensive fruiting‐trait dataset for 88 species spanning all eight genera of the family. Using this framework, we reconstructed the evolutionary history of prolonged delayed fertilization underlying the 2‐year fruiting strategy and quantified the timing, frequency, and direction of transitions between 1‐ and 2‐year fruiting. We further tested whether shifts in fruiting strategy are evolutionarily associated with two hypothesized constraints—pollination mode and leaf habit. Together, these analyses provide a framework for reconstructing the evolutionary history of 2‐year fruiting in the Fagaceae, including its timing and frequency of origin and loss, and for evaluating whether shifts in fruiting strategy have been evolutionarily coordinated with changes in pollination mode or leaf habit.

## 2 Materials and Methods

### 2.1 Trait Data Collection

We followed the taxon‐sampling scheme of Zhou et al. (2022), which includes 90 Fagaceae species across eight genera and provides a genome‐scale nuclear phylogeny. The extant Fagaceae comprise approximately 900–1000 species across these genera (Deng et al. 2022; Kremer et al. 2012; Satake and Kelly 2021). Although this sampling represents a subset of the total species diversity, it captures all major lineages within the family and is currently the most comprehensive dataset available for comparative analyses across all genera. To maintain consistency with recent molecular phylogenetic frameworks (Hipp et al. 2020; Liu et al. 2025; Shen et al. 2025; Yang et al. 2025; Zhou et al. 2022), we adopted updated generic circumscriptions: species listed as *Cyclobalanopsis* and *Formanodendron* in the eFlora of China were treated as *Quercus* and *Trigonobalanus*, respectively (Brach and Song 2006), and species listed as *Lithocarpus* in the eFlora of North America were treated as *Notholithocarpus* (Nixon 1997). We excluded *Quercus liaotungensis* Koidz. as it is treated as a synonym of *Q. mongolica* Fisch. ex Ledeb. in eFlora of China (Brach and Song 2006), which was already included in the dataset.

We compiled fruiting trait data–1‐ or 2‐year fruiting–from the eFlora of China (Brach and Song 2006) and the eFlora of North America (Nixon 1997) (Table S1), for 88 species encompassing all eight genera. For 19 species for which information on the three traits was unavailable or potentially inaccurate in the eFlora of China or the eFlora of North America, we consulted additional references (listed in Table S1). For eight species for which information was unavailable from the literature, we examined online herbarium specimens from the herbarium databases listed in Table S2. Notably, *Lithocarpus tephrocarpus* (Drake) A.Camus was classified as a 2‐year fruiting species based on field observations. Traits for pollination mode (anemophily or entomophily) and leaf habit (deciduous or evergreen) were also compiled based on the references listed in Table S1. In this study, leafing traits were classified into two categories: deciduous and evergreen. However, four species have been reported to exhibit an intermediate condition, often referred to as “subevergreen” (also recorded as brevideciduous and semi‐evergreen). In such cases, assigning three states to a single trait would prevent the implementation of discrete models in BayesTraits (Pagel et al. 2004; Pagel and Meade 2006). In Hipp et al. (2018), which provides a detailed assessment of leaf phenology in oaks, *Quercus engelmanii* Greene, *Q. fusiformis* Small, and *Q. virginiana* Mill were classified as brevideciduous, defined as “leaves generally present year‐round or with only a brief period of leaflessness.” Given that leaves are present for most of the year, we treated these species as distinct from deciduous and assigned them to the evergreen category for our binary classification. For *Q. castaneifolia* C.A.Mey, we consulted additional references (see Table S1), all of which consistently classify this species as deciduous; we therefore treated it as a deciduous species.

Using specimen images from online herbarium databases, we first confirmed species identities based on diagnostic morphological traits and then determined whether each species exhibits 1‐ or 2‐year fruiting. For each specimen, we examined twigs bearing female reproductive organs. Current‐year and previous‐year twigs were distinguished using leaf scars as morphological markers. To classify fruiting traits, we treated all duplicate specimens derived from the same collection event as a single specimen group. A species was classified as 2‐year fruiting if at least one specimen group contained two distinct developmental stages of female reproductive organs: (i) flowers or fruits retained from the previous year and (ii) flowers produced in the collection year. Species that did not meet this criterion were classified as 1‐year fruiting (see Note in Table S3). Because individual herbarium sheets often include only a single developmental stage, evaluating specimen groups rather than individual sheets enabled us to detect cases in which two stages occurred among duplicates from the same collection event. We assumed that flowers originating from a single flowering event develop within the same season; therefore, the coexistence of two clearly distinct developmental stages within a specimen group was interpreted as evidence for 2‐year fruiting. *Trigonobalanus excelsa* Lozano, Hern.Cam. et Henao, for which reliable information could not be obtained, even after examination of herbarium specimens, was excluded from subsequent analyses.

### 2.2 Phylogenetic Reconstruction

**FIGURE A1:**
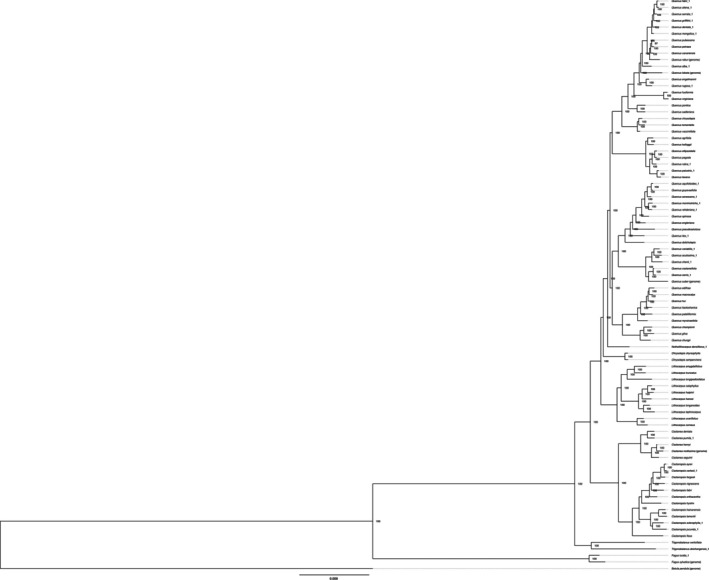
Majority‐rule consensus tree reconstructed with MrBayes v.3.2.7 from the nuclear genome dataset of Zhou et al. (2022), after removing excluded samples. Posterior probabilities (PP) are shown at each node. Scale bar indicates substitutions per site.

We conducted a Bayesian phylogenetic analysis using MrBayes v3.2.7 (Ronquist et al. 2012) using nucleotide alignments of 2124 nuclear genes provided by Zhou et al. (2022). Sequences not belonging to our focal taxa (Table S1) were excluded. We ran four independent Markov chain Monte Carlo (MCMC) analyses, each for 1,000,000 generations with sampling every 500 generations. The first 25% of sampled trees from each run were discarded as burn‐in. The remaining post‐burn‐in trees were pooled to generate a 50% majority‐rule consensus tree and to calculate posterior probabilities for nodes (Figure A1).

The model settings followed Zhou et al. (2022), adopting the optimal partitioning scheme and nucleotide substitution models using PartitionFinder 2 v1.1 (Lanfear et al. 2017) under the Akaike Information Criterion (AIC) (Akaike 1974). To account for topological uncertainty in subsequent ancestral character state reconstructions, we randomly resampled 500 trees from the post‐burn‐in trees.

### 2.3 Reconstructing Ancestral States and Testing Correlated Evolution

We applied BayesTraits v3.0.5 (Pagel et al. 2004; Pagel and Meade 2006) to infer the ancestral states of fruiting types, pollination modes, and leaf habits using the multistate model, and to test for correlated evolution among these traits using the discrete model. The multistate model allows the reconstruction of evolutionary changes in traits that exist in a finite set of discrete conditions on phylogenies, facilitating both ancestral state reconstruction and hypothesis testing of evolutionary models (Pagel et al. 2004). The discrete model is used to test whether two discrete binary traits have evolved in a correlated manner by comparing dependent and independent evolutionary scenarios (Pagel 1994; Pagel and Meade 2006). To satisfy the requirements of the discrete model, we encoded traits as follows: the fruiting trait (1‐year fruiting = 0, 2‐year fruiting = 1), the pollination mode (anemophily = 0, entomophily = 1), and the leafing trait (deciduous = 0, evergreen = 1) (see Table S1).

**FIGURE A2:**
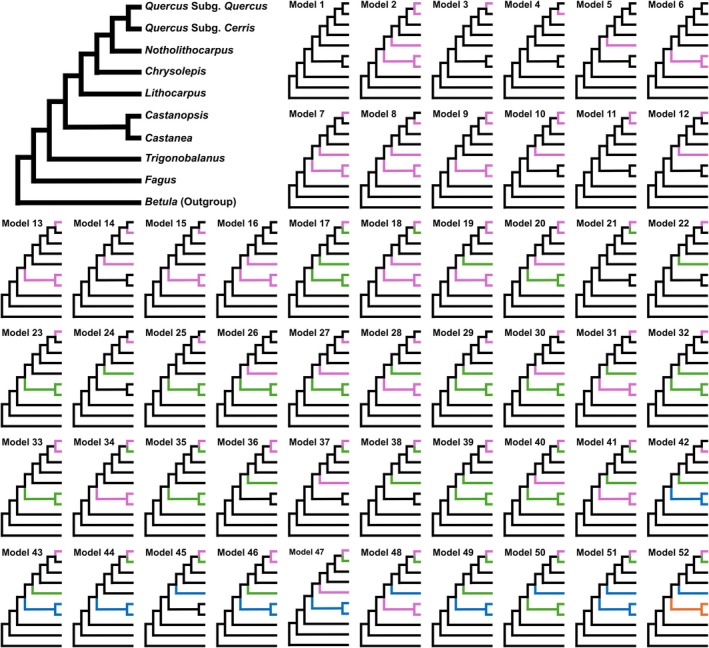
Overview of the 52 alternative model settings considered in the multistate model analysis of fruiting traits. Model IDs are shown at the upper left of each tree, and branches assigned different transition rates are indicated in various colors.

Within the multistate model framework, we compared an all‐rates‐different model (ARD), which estimates distinct transition rates among all states, with an equal‐rates model (ER) that constrains all transitions to be identical, using the Restrict function in BayesTraits. For fruiting traits, we additionally fitted clade‐heterogeneous variants in which transition rates were allowed to differ among major clades, given substantial genus‐level variations in the prevalence of fruiting traits (Satake and Kelly 2021). To test this, we used the AddPattern function in BayesTraits to assign clade‐specific transition rates to four specie‐rich clades (each ≥ 10 species): *Quercus* subg. *Quercus*, *Quercus* subg. *Cerris*, *Lithocarpus*, and *Castanea* + *Castanopsis* (Figure 1B). This resulted in 52 alternative model specifications (Figure A2). Furthermore, several *Quercus* species exhibit intraspecific or even intra–individual polymorphism in fruiting type (e.g., *Q. suber* L.: Díaz‐Fernández et al. 2004; Elena‐Rossello et al. 1993). For *Q. suber* , we therefore conducted ancestral state reconstructions under two alternative assumptions, treating the species as 1‐ or 2‐year fruiting; these were analyzed as separate datasets (“Fruiting_A” and “Fruiting_B”; Table S4). For pollination mode and leaf habit, we compared only the equal‐rates (ER) and all‐rates‐different (ARD) models (i.e., no clade‐heterogeneous variants).

**FIGURE A3:**
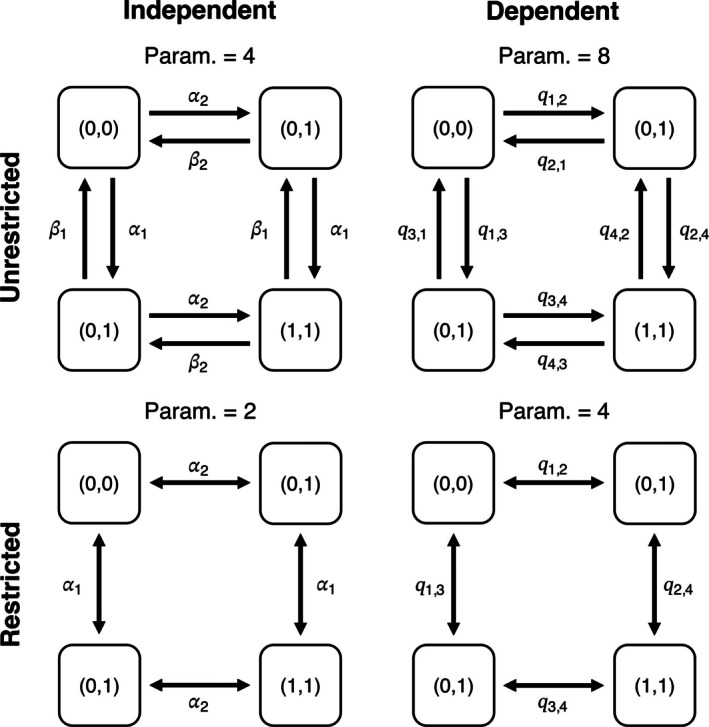
Schematic representation of four different model configurations in the discrete model analyses. In the independent model (left), each trait evolves separately with transition rates α and β, resulting in four parameters (unrestricted) or two parameters (restricted). In the dependent model (right), the two traits evolve jointly with transition rates q, resulting in eight parameters (unrestricted) or four parameters (restricted). Boxes indicate possible combined states of the two traits, and arrows represent allowed transitions with associated rate parameters.

In the discrete model (Pagel 1994), we compared four alternative models of trait evolution to test for correlated evolution between fruiting traits and other traits, including pollination and leafing traits: (i) an independent ARD model (4 parameters), (ii) an independent ER model (2 parameters), (iii) a dependent ARD model (8 parameters), and (iv) a dependent ER model (4 parameters) (Figure A3). In the independent model, the two binary traits evolve independently along separate continuous‐time Markov chains, indicating that changes in one trait occur regardless of the state of the other. In the dependent model, the transition rates are state‐dependent, such that the probability of a change in one trait depends on the state of the other. This reflects a scenario in which the evolution of one trait may influence, constrain, or be coordinated with the evolution of the other.

Model selection was performed using Bayes factors (BF) calculated from marginal likelihoods of competing models (Kass and Raftery 1995). We computed 2lnBF, defined as $2\ln\;\mathrm{BF}=2\left[\ln\;m\left(\text{complex}\right)-\ln\;m\left(\text{simple}\right)\right]$, where $m\left(\cdot \right)$ denotes the marginal likelihood. The resulting 2lnBF values were interpreted following Kass and Raftery (1995): positive, strong, and very strong (> 10) evidence in favor of the more complex model.

All BayesTraits analyses were run for 1,250,000 iterations, with the first 250,000 discarded as burn‐in and sampling performed every 1000 iterations. At each step of the MCMC runs, one tree was randomly chosen from a posterior sample of 500 resampled post‐burn‐in trees to integrate topological and branch‐length uncertainty. Marginal likelihoods were estimated using the stepping‐stone sampler (Xie et al. 2011) with 100 stones and 10,000 iterations per stone; model‐specific values are reported in Table S4. We placed an exponential hyperprior on transition rate parameters, with the mean drawn from a uniform distribution between 0 and 100, and used reversible‐jump MCMC to traverse model space (Pagel and Meade 2006; Plummer et al. 2006). Convergence of MCMC chains for each model was assessed using the R package “coda”, and effective sample sizes (ESS) for all fluctuating parameters exceeded 200. Posterior state probabilities were then mapped onto a 50% majority‐rule consensus tree. We time‐scaled with eight fossil calibrations (age ranges following Zhou et al. 2022) using the chronos function in the R package ape (Paradis et al. 2004; Paradis and Schliep 2019) to add the simplified time axis.

## 3 Results

### 3.1 Ancestral Character State Reconstruction

#### 3.1.1 Estimated Evolutionary History of the Fruiting Traits

**FIGURE 2:**
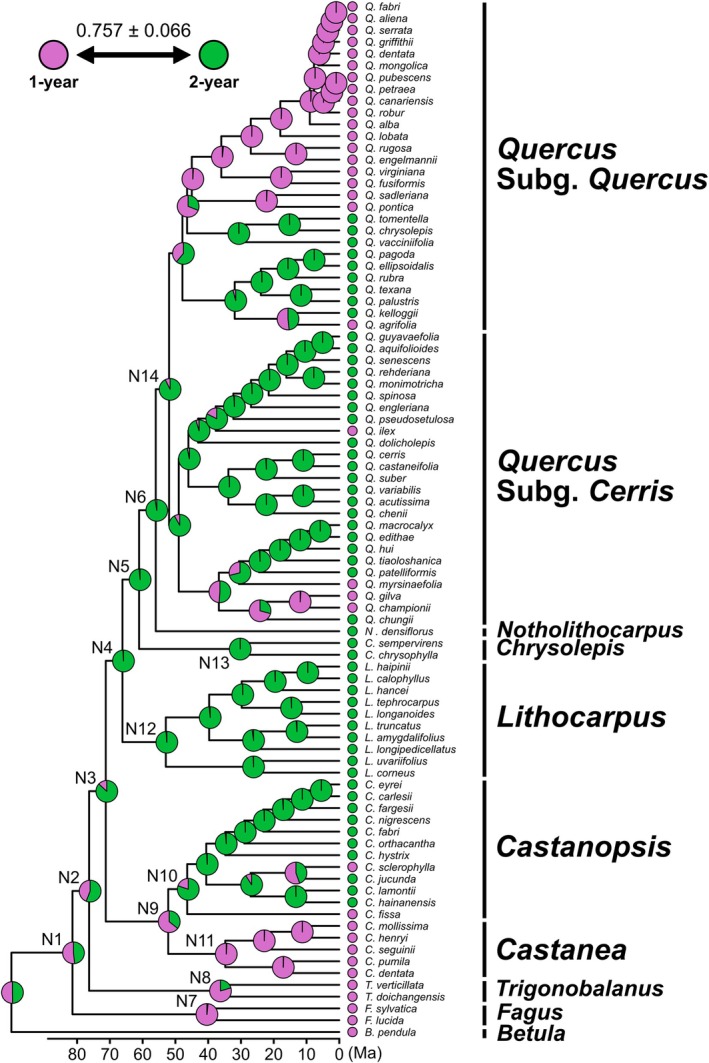
Estimated evolutionary history of fruiting traits based on the best‐supported result from the multistate model analyses when *Quercus suber* is treated as a 2‐year fruiting. Pink and green represent 1‐ and 2‐year fruiting, respectively. The pie chart at each node within the phylogenetic tree represents the proportional probability of 1‐ and 2‐year fruiting. The value below the phylogenetic tree indicates the relative transition rate between the 1‐ and 2‐year fruiting.

The best‐fit model for ancestral state reconstruction for the fruiting trait was the single transition rate model (Table S4), in which transitions between 1‐ and 2‐year fruiting occurred at equal rates across the tree (0.757 ± 0.066; Figure 2). Under this model, node N3—uniting the six clades except *Fagus* and *Trigonobalanus*—was strongly supported as the 2‐year fruiting (86.8% ± 2.56%; Figure 2; Table S5). In contrast, the ancestral state of the deeper nodes N1 and N2, corresponding to the early divergences involving *Fagus* and *Trigonobalanus*, could not be confidently resolved (Figure 2), reflecting limited resolution at the base of the lineage.

The 2‐year fruiting state was consistently retained after the divergence of *Lithocarpus*, *Chrysolepis*, and *Notholithocarpus* at node N4‐6, each supported by posterior probabilities exceeding 95% (Figure 2; Table S5). Within the clade of *Castanea* and *Castanopsis*, the ancestral node N9 was equivocal, with a posterior probability of 64.3% ± 4.76% marginally supporting the 1‐year fruiting. In contrast, the common ancestors of all other genera containing the 2‐year fruiting species were inferred to be the 2‐year fruiting, with strong posterior support (*Castanopsis*: 80.2% ± 1.52% at node N10; *Lithocarpus*: 98.7% ± 1.06% at “N12”; *Chrysolepis*: 99.9% ± 0.0702% at node N13; *Quercus*: 92.6% ± 4.45% at node N14; Table S5). Conversely, the ancestors of genera containing only the 1‐year fruiting were inferred to be the 1‐year fruiting (*Fagus*: 98.3% ± 1.12% at node N7; *Trigonobalanus*: 79.6% ± 8.19% at node N8; *Castanea*: 99.2% ± 0.602% at node N11; Table S5).

**FIGURE A4:**
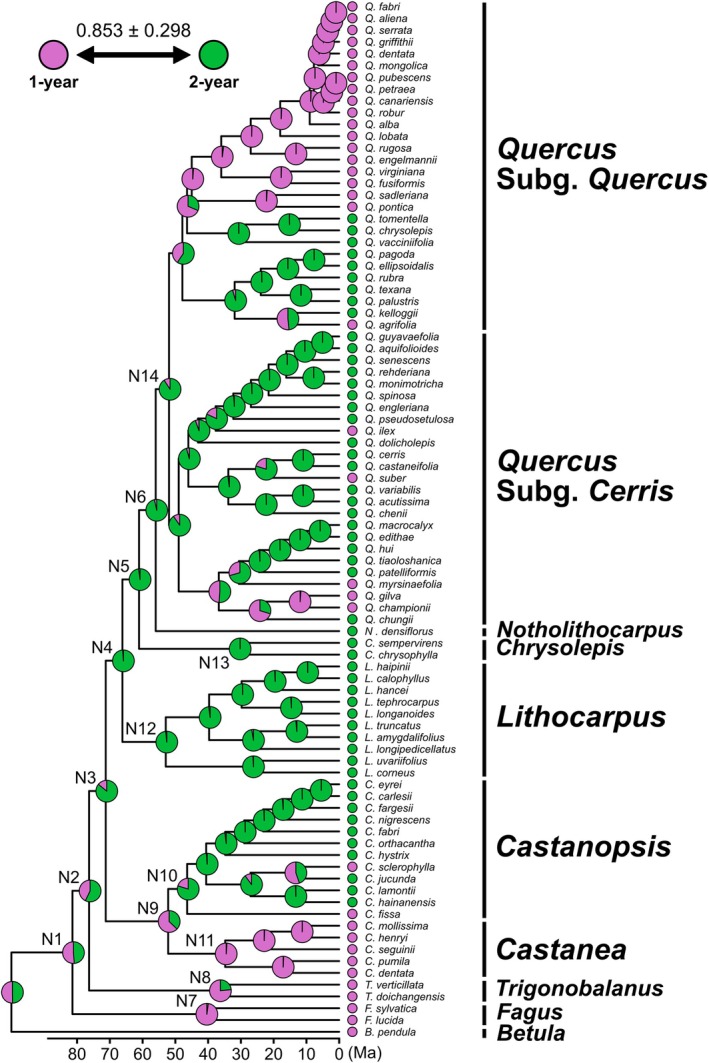
Estimated evolutionary history of fruiting traits based on the best‐supported result from the multistate model analyses when *Quercus suber* is treated as a 1‐year fruiting. Pink and green represent 1‐ and 2‐year fruiting, respectively. The pie chart at each node within the phylogenetic tree represents the proportional probability of 1‐ and 2‐year fruiting. The value below the phylogenetic tree indicates the relative transition rate between the 1‐ and 2‐year fruiting.

Our analyses also showed that transitions from the 2‐year to the 1‐year fruiting occurred multiple times independently across lineages. The 1‐year fruiting species arose predominantly from the 2‐year fruiting clades in *Castanopsis*, *Quercus* subg. *Cerris* and subg. *Quercus*. Notably, the common ancestor of Sects. *Ponticae*, *Virentes*, and *Quercus* within subg. *Quercus* was reconstructed with a posterior probability of 99.2% ± 0.644% for the 1‐year fruiting, consistent with the shared state across these sections. In addition, the analysis treating *Quercus suber* as a 1‐year fruiting species yielded results consistent with those described above (Figure A4).

#### 3.1.2 Estimated Evolutionary History of the Pollination Traits

**FIGURE 3:**
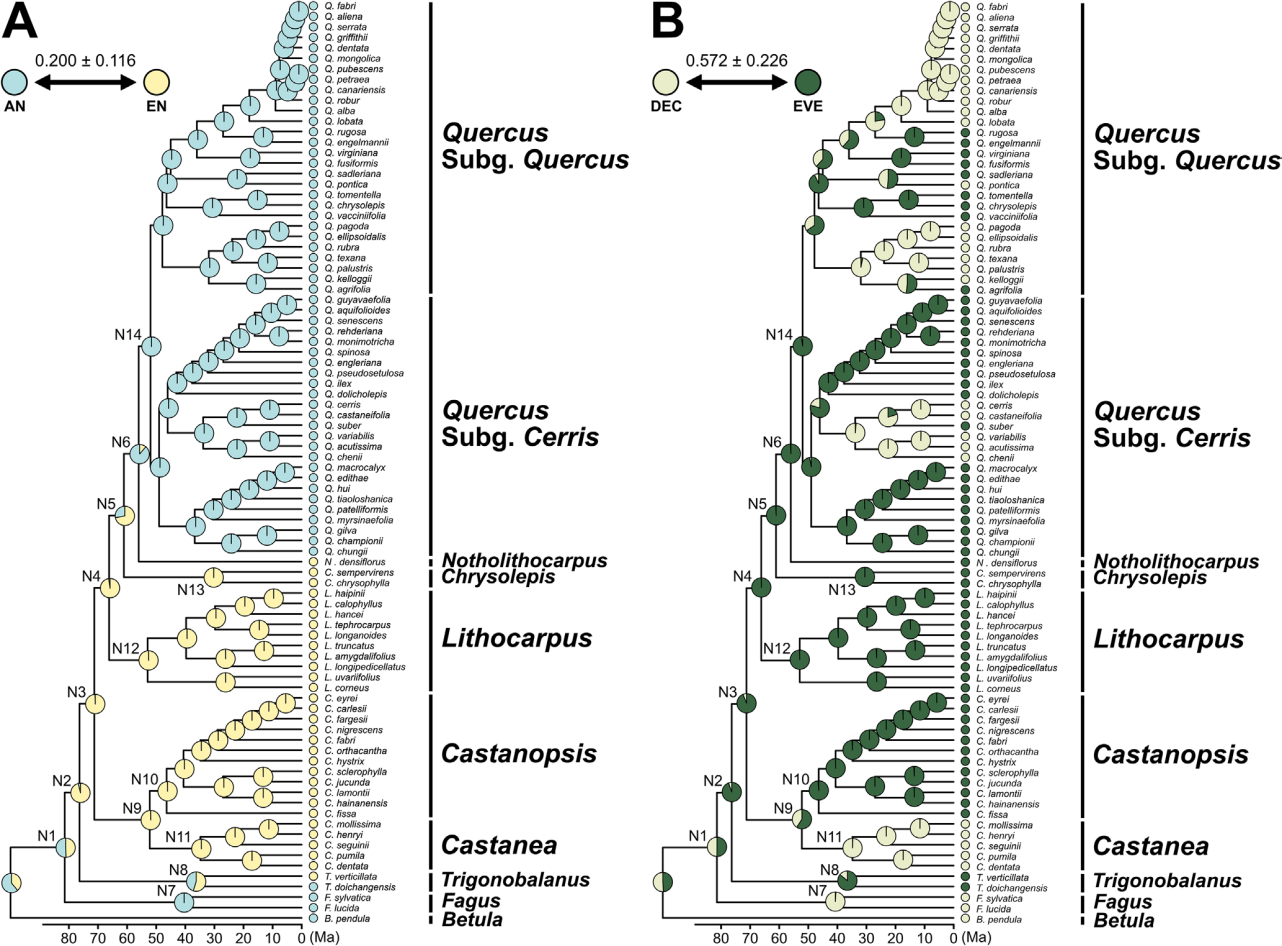
Estimated evolutionary history of the traits potentially associated with prolonged delayed fertilization, based on the best‐supported results from the multistate model analyses. The pie chart at each node within the phylogenetic tree represents the proportional probability of alternative states, and the values below the phylogeny indicate the relative transition rates between states. (A) Ancestral state reconstruction of the pollination trait. Light blue and yellow represent anemophily (AN) and entomophily (EN), respectively. (B) Ancestral state reconstruction of the leafing trait. Light brown and dark green represent deciduous (DEC) and evergreen (EVE), respectively.

Multistate analyses for pollination mode showed that a model constraining transition rates was selected (Table S4). The estimated relative transition rate was 0.200 ± 0.116 (Figure 3A). For pollination mode, the node N2 comprising *Trigonobalanus* and its sister clade was strongly supported as entomophilous with a posterior probability of 96.3% ± 2.46%, although the ancestral state of Fagaceae was ambiguous (Figure 3A). This state was retained until the divergence of *Lithocarpus*, after which the common ancestor of *Notholithocarpus* and *Quercus* at the node N6 was strongly supported as anemophilous (88.9% ± 4.13%). Independent origins of anemophily were further supported in *Fagus*, *Trigonobalanus doichangensis*, and *Quercus* (Figure 3A).

#### 3.1.3 Estimated Evolutionary History of the Leafing Traits

For leaf habit, a model with constrained transition rates was selected (Table S4), and the estimated relative transition rate was 0.572 ± 0.226 (Figure 3B). The ancestral state of Fagaceae was likewise ambiguous, as indicated by node N1, which showed comparable likelihoods for deciduous and evergreen states (Figure 3B). However, the node N2 comprising *Trigonobalanus* and its sister clade was strongly supported as evergreen (94.5% ± 4.70%). The common ancestors of the evergreen genera were supported as evergreen with posterior probabilities exceeding 85.9% (*Trigonobalanus*: 85.9% ± 7.59% at node N8; *Castanopsis*: 98.7% ± 1.07% at node N10; *Lithocarpus*: 99.3% ± 0.716% at node N12; *Chrysolepis*: 99.9% ± 0.0507% at N13; *Quercus*: 97.5% ± 1.68% at node N14; Figure 3B), indicating that deciduousness observed in *Castanea* and some lineages of *Quercus* is a derived condition. Because the ancestral state of Fagaceae remains unresolved, it is not possible to determine whether the deciduous habit of *Fagus* represents an ancestral or derived state.

### 3.2 Testing Correlated Evolution Between Two Traits

**FIGURE 4:**
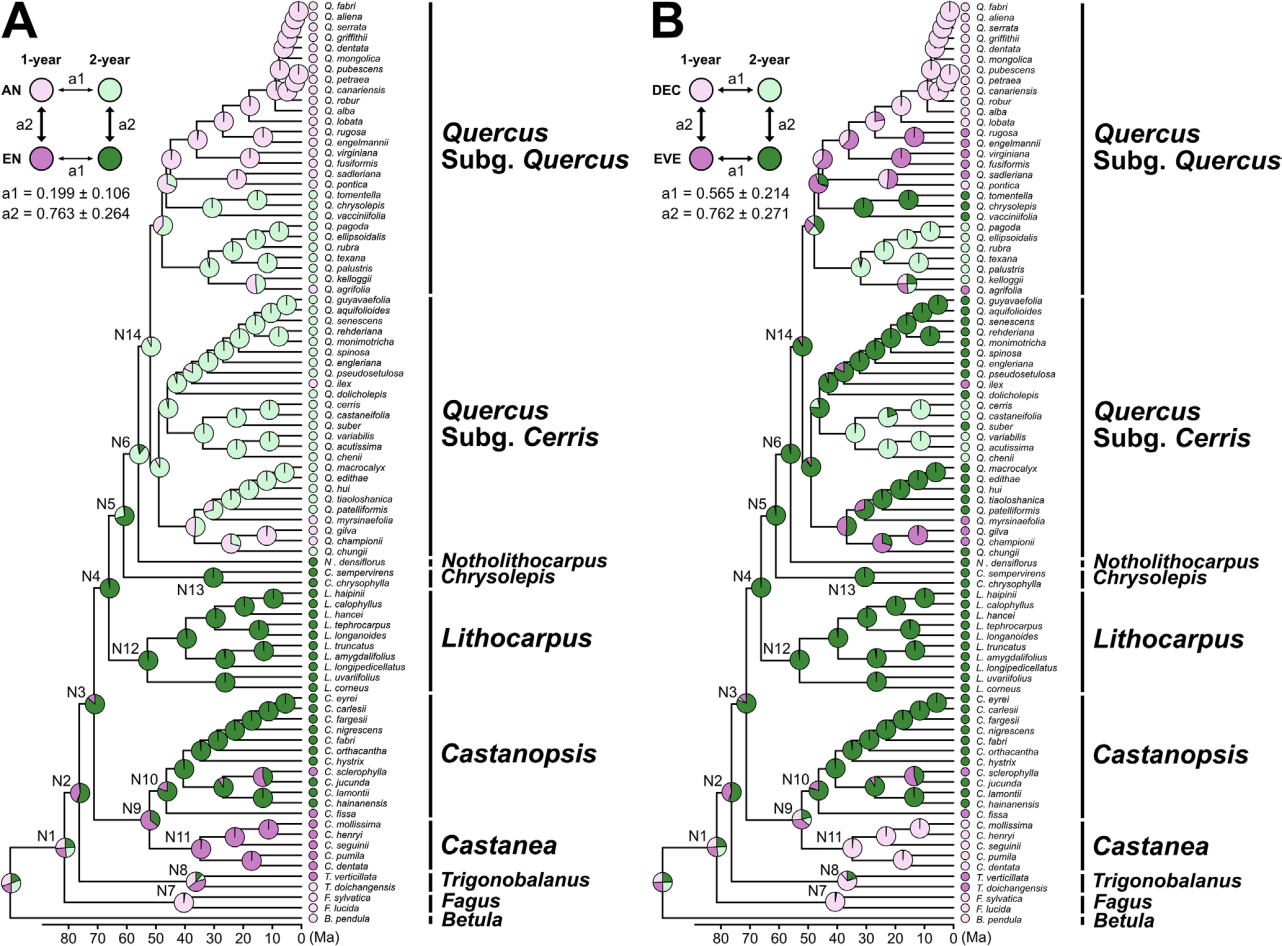
Estimated evolutionary history of the correlated evolution between fruiting trait and (A) pollination trait and (B) leafing trait, based on the best‐supported result from the discrete model analyses. The pie chart at each node within the phylogenetic tree represents the proportional probability of the four states. The values below the phylogenetic tree indicate the relative transition rates among the four states. (A) Ancestral state reconstruction of the fruiting and pollination traits. The four states are represented by light pink, dark pink, light green, and dark green, corresponding to 1‐year fruiting (1‐year) and anemophily (AN), 1‐year fruiting and entomophily (EN), 2‐year fruiting (2‐year) and anemophily, and 2‐year fruiting and entomophily, respectively. (B) Ancestral state reconstruction of the fruiting and leafing traits. The four states are represented by light pink, dark pink, light green, and dark green, corresponding to 1‐year fruiting and deciduous (DEC), 1‐year fruiting and evergreen (EVE), 2‐year fruiting and deciduous, and 2‐year fruiting and evergreen, respectively.

Finally, we examined whether fruiting traits evolved in correlation with pollination or leafing traits. Based on likelihood ratio tests, the independent ER model was selected as the best‐fitting model for both trait combinations (Figure 4; Table S4), suggesting that there is no support for correlated trait evolution. In both analyses, transitions in fruiting, pollination, and leafing traits occurred independently, with estimated transition rates closely matching those obtained when ancestral states were reconstructed independently for each trait (Figures 2, 3, 4; Figure A4).

## 4 Discussion

Our ancestral state reconstructions indicate that the 2‐year fruiting originated once in the most recent common ancestor of the genera in which this trait is present today (node N3 uniting the six clades except *Fagus* and *Trigonobalanus*: see Figure 2; Figure A4). Previous molecular dating studies have placed the emergence of this lineage in the late Cretaceous to early Paleocene (approximately 85–60 Ma; Liu et al. 2025; Yang et al. 2025; Zhou et al. 2022). Because this timeframe coincides with major global climate changes and the emergence of seasonality (Toumoulin et al. 2022), it is tempting to speculate that these environmental changes may have facilitated—or driven—the evolution of delayed fertilization and the resulting 2‐year fruiting through the occurrence of periods unsuitable for reproduction. A more detailed understanding of this evolutionary context will require fossil‐informed biogeographic analyses that explicitly link environmental dynamics with the origin and diversification of prolonged delayed fertilization.

Following the initial acquisition of the 2‐year fruiting, multiple independent reversions to the 1‐year fruiting were inferred across the family. These reversions occurred in the common ancestor of *Castanea*, within *Castanopsis*, and in several clades of *Quercus*, indicating that although shifts back to 1‐year fruiting are relatively rare, they have arisen repeatedly across different genera. Additional cases are likely present in *Lithocarpus*, where several species not included in our analysis (e.g., *L. dodonaeifolius* (Hayata) Hayata, *L. formosanus* (Skan) Hayata) are known to exhibit 1‐year fruiting (Brach and Song 2006). Notably, no cases of reversion of the 2‐year fruiting were detected in any lineage that had reverted to the 1‐year fruiting lineages. A prominent example involves the common ancestor of *Quercus* Sects. *Ponticae*, *Virentes*, and *Quercus*, which arose during the Eocene (56–33.9 Ma). Although this ancestor is inferred to have undergone a transition back to the 1‐year fruiting, its subsequent range expansion across Eurasia and North America was not accompanied by a secondary gain of the 2‐year fruiting (Figure 2; Figure A4; Denk et al. 2017; Hipp et al. 2018, 2020; Zhou et al. 2022). Likewise, in *Castanea*, all extant species are 1‐year fruiting without any reversion to 2‐year fruiting.

In *Quercus suber* , both 1‐ and 2‐year fruiting have been reported to coexist within a species (Elena‐Rossello et al. 1993; Díaz‐Fernández et al. 2004). Díaz‐Fernández et al. (2004) showed that individuals with later flowering phenology tend to exhibit a higher proportion of 2‐year female flowers across multiple sites with contrasting environmental conditions. They also reported that the proportion of individuals with a 2‐year fruiting increases toward higher latitudes. These findings suggest that when delayed flowering constrains the time available to complete fruit development within a single season, the 2‐year fruiting may be favored. This pattern is consistent with theoretical predictions and observed flowering and fruiting phenology (Satake and Kelly 2021; Araye et al. 2023), which suggests that species flowering closer to unfavorable seasons are more likely to exhibit 2‐year fruiting.

Ancestral‐state reconstructions strongly support entomophily and evergreen leaf habit as ancestral traits of Quercoideae (node N2; Figure 3) predating the emergence of the 2‐year fruiting strategy at the most recent common ancestor (node N3). This pattern indicates that 2‐year fruiting evolved against a background of entomophilous pollination and evergreen leaf habit. Insect pollination characterizes extant genera such as *Castanea*, *Castanopsis*, *Lithocarpus*, *Chrysolepis*, *Notholithocarpus*, and *Trigonobalanus verticillata*, which possess compound inflorescences and attract generalist insect pollinators (Chen et al. 2025; Crepet and Nixon 1989; Larue and Petit 2024; Larue et al. 2021; Kaul and Abbe 1984; Kaul 1986; Nixon and Crepet 1989; Petit and Larue 2022; Sadowski et al. 2020; Soepadmo 1972; Wright and Dodd 2013; Yuan et al. 2024), whereas wind pollination in *Quercus* and *Trigonobalanus doichangensis* represents derived states (Manos et al. 2001; Nixon and Crepet 1989; Zhou et al. 2022); for *Fagus*, the ancestral or derived status of wind pollination remains unresolved, despite previous suggestions of wind pollination as ancestral in Fagales and Fagaceae (Stephens et al. 2023; Yao et al. 2023).

Evergreen leaf habit, likely acquired under warm Cretaceous–Paleocene climates, has been phylogenetically conserved in low‐latitude Asian lineages such as *Castanopsis*, *Lithocarpus*, and some sections of *Quercus* (Sects. *Cyclobalanopsis* and *Ilex*), whereas independent transitions to deciduousness accompanied northward range expansion in *Castanea* and several *Quercus* lineages (Sects. *Lobatae* and *Quercus*), likely driven by colder and more seasonal climates (Barrón et al. 2017; Cavender‐Bares 2018; Deng et al. 2018; Fontes et al. 2025; Hipp et al. 2018; Jiang et al. 2019; Kikuzawa 1991; Sancho‐Knapik et al. 2021; Soepadmo 1972; Zanne et al. 2014). Multiple secondary transitions from deciduous to evergreen states further indicate that leaf habit evolution reflects both phylogenetic conservatism and repeated adaptive shifts (Furze et al. 2021; Hipp et al. 2018; Sancho‐Knapik et al. 2021).

Despite these pronounced evolutionary dynamics, our discrete‐trait analyses detected no evidence for correlated evolution between fruiting strategy and either pollination mode or leaf habit. While the number of transitions inherently limits statistical power, this result suggests no strong coupling between these traits.

In addition to pollination mode and leaf habit, the resource investment cost associated with fruit development may represent an important factor influencing the evolution of the 2‐year fruiting. Allocation to fruit tissues could impose substantial physiological constraints, potentially shaping reproductive timing. Previous theoretical study (Satake and Kelly 2021) predicts that delayed fertilization is more likely to evolve when the time required for seed maturation—closely linked to investment cost—is prolonged, suggesting a mechanistic link between developmental duration and the evolution of 2‐year fruiting. However, empirical evidence from extant species remains inconclusive. Available data indicate that the amounts of carbon, nitrogen, and phosphorus invested in cupules and fruits do not consistently differ between 1‐ and 2‐year fruiting species (Wang et al. 2022). Furthermore, some species producing relatively large fruits still exhibit 1‐year fruiting (Wang et al. 2022), indicating that investment cost alone is insufficient to explain the evolution of the 2‐year fruiting. These observations highlight the need for a more integrative approach to fully understand the evolutionary drivers for the evolution of the 2‐year fruiting.

At the same time, integrating such ultimate drivers with proximate molecular mechanisms will be essential for a comprehensive understanding of delayed fertilization. Emerging genomic and transcriptomic approaches now make it possible to investigate how developmental processes are regulated under natural environmental conditions (Satake et al. 2022, 2023; Kudo et al. 2025; Lara‐De La Cruz and Chávez‐Vergara 2026). For instance, genes governing ovule developmental timing and regulatory pathways involving hormonal control of dormancy and its release may provide key mechanistic links between seasonal environments and reproductive timing. Future studies that combine comparative phylogenetic analyses with molecular and developmental investigations will therefore be crucial for elucidating the evolutionary processes underlying delayed fertilization in the Fagaceae.

## Author Contributions

**Takenori Shagawa:** data curation (lead), formal analysis (equal), validation (equal), visualization (equal), writing – original draft (lead), writing – review and editing (equal). **Chihiro Myotoishi:** data curation (supporting), formal analysis (equal), methodology (equal), validation (equal), visualization (equal). **Tetsukazu Yahara:** data curation (supporting), supervision (supporting), writing – review and editing (equal). **Ryosuke Imai:** methodology (equal). **Min Deng:** data curation (supporting). **Akiko Satake:** conceptualization (lead), funding acquisition (lead), methodology (equal), project administration (lead), supervision (lead), validation (equal), writing – review and editing (equal).

## Funding

This work was supported by the Japan Society for the Promotion of Science (JP23H04965 and JP23H04966).

## Conflicts of Interest

The authors declare no conflicts of interest.

## Supporting information


**Table S1:** List of Fagaceae species analyzed and their trait information.

**Table S2:** List of herbaria and corresponding data portals for cited Fagaceae specimens.

**Table S3:** Online herbarium specimens used for data collection.

**Table S4:** Summary of model settings and results of ancestral state reconstruction using BayesTraits. The model shown in bold was selected as the best‐fitting model based on Bayes factor comparisons calculated from log marginal likelihoods.

**Table S5:** Probability of trait states at nodes N1–N14 under the best‐fit multistate models for each analyzed trait dataset.

## Data Availability

Trait data used in this study are publicly available from the eFloras of China (Brach and Song 2006) and the eFlora of North America (Nixon 1997). Additional literature sources used for trait data compilation are listed in Table S1. Information on digitized herbarium specimens used to verify and refine trait assignments is provided in Tables S2 and S3.
